# Supplementary material for: Characterization of Staphylococcus aureus Isolates from Bovine Mastitis and Bulk Tank Milk: First Isolation of Methicillin-Susceptible Staphylococcus aureus in Japan
Source: Microorganisms. 2022 Oct 26;10(11):2117. doi: 10.3390/microorganisms10112117 (PMC9696108; doi:10.3390/microorganisms10112117)
Supplement: Supplementary file 1 [file microorganisms-10-02117-s001.zip › Supplementary Figure S1.docx]

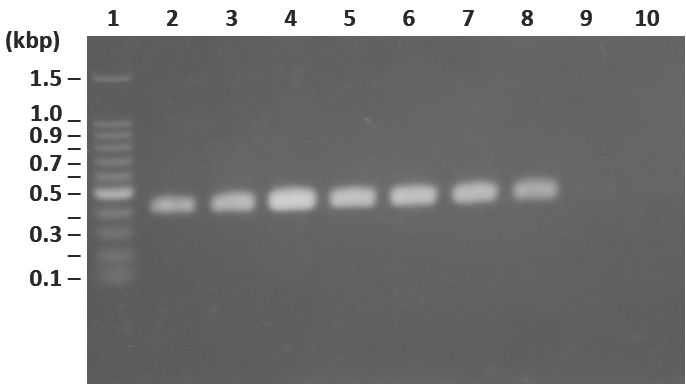


**Supplementary Figure S1.** Identification of tetracycline resistance genes via PCR analysis in *S. aureus* strains isolated from mastitis cows. Lane 1, marker; lanes 2–8, CC398 isolate clones (2, SA4; 3, SA21; 4, SA22; 5, SA23; 6, SA27; 7, SA39; 8, SA58); lane 9, CC705 isolate SA59; lane 10, CC97 isolate SA10.
